# Perinatal Programming of Childhood Asthma

**DOI:** 10.1155/2012/438572

**Published:** 2012-08-30

**Authors:** Kuender D. Yang

**Affiliations:** ^1^Department of Pediatrics, Show Chwan Memorial Hospital, Changhua 500, Taiwan; ^2^Central Taiwan University of Science and Technology, Taichung 40601, Taiwan

## 1. Developmental Origin of Childhood Asthma

Prevalence of childhood asthma has worldwide increased in recent decades. Different genome-wide studies have identified that more than 100 genes in 22 chromosomes were associated with asthma. Different genetic backgrounds in different environments might modulate the susceptibility of asthma. This has been attributed to industrialized environment such as air pollution and microbial-deprivation ecology that polarize the immune response towards allergy sensitization in perinatal stage. Recently, evidence has shown that allergy sensitization may occur in fetal life, and influence of fetal environment may cause epigenetic programming of diseases in adults. Apparently, asthma is not an exception from the Developmental Origins of Health and Diseases (DOHaD), in which both the pre- and postnatal environments could shape the developmental programming of asthma developed in infancy, childhood, and even adulthood.

The association between prenatal environment and disease risk in adults is first demonstrated by Barker showing low birth weight was linked to ischemic heart disease in adult life in 1986 [1], and collaborating with Hales to raise the thrifty phenotype hypothesis emphasizing the importance of developmental plasticity in type 2 diabetes mellitus in 1992 [2]. They also proved that obesity in adults could be traced back to prenatal exposure to famine in the Dutch hunger winter of War World II [3]. Now the prenatal programming of diseases in adults have been linked to a number of chronic diseases including metabolic syndrome, type 2 diabetes, hypertension, cardiovascular disease, schizophrenia, osteoporosis, overweight/obesity, and asthma. Taken together, this suggests that childhood asthma, although heritable, is significantly affected by different environments in perinatal stage.

## 2. Risk to and Protection from Perinatal Programming of Childhood Asthma

**Figure 1:**
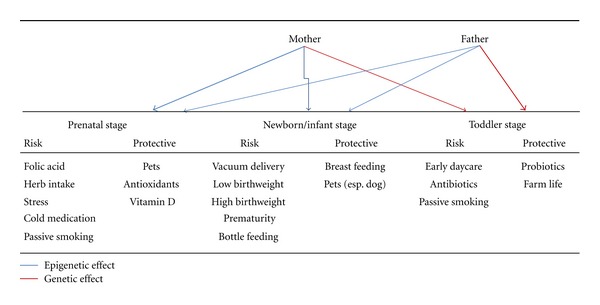
Summary of environmental (epigenetic) and genetic programming of asthma.

In this special issue, we included 8 papers depicting effects and potential mechanisms of perinatal environments including intrauterine growth trajectory, maternal exposure of cold and herb medication, perinatal exposure to pets, perinatal gut microbiota, bottle feeding, genetic determinant of cockroach allergy, interaction of parental atopy and genes, and gene-environment interactions on the development of asthma. These papers together have highlighted different epigenetic and genetic effects of perinatal environments and parental genetic backgrounds on the perinatal programming of asthma. As summarized from these articles and shown in Figure 1, maternal (prenatal) environments have a strong impact on the programming of childhood asthma in which folic acid supplement, parental smoking, oxidative stress, and cold and herb medication are risk factors to childhood asthma. In newborn and infant stage, prematurity, vacuum delivery, skewed (high or low) birthweight, and bottle feeding are risk factors to childhood asthma. In toddler stage, early daycare placement, antibiotic uses, and parental smoking are also associated with childhood asthma. The environmental programming of asthma in perinatal stage is likely mediated via epigenetic modification or immune differentiation toward a higher T-cell type 2 (Th2) response or a lower Treg differentiation. For instance, we found that maternal atopy interacting with the CTLA-4 gene polymorphisms for antenatal IgE production begins in prenatal stage [4], and miRNA-21 underexpression in neonatal leukocytes, which is correlated to overexpression of TGFR expression, is associated with allergic rhinitis in childhood [5]. Recently, our preliminary studies on prenatal tobacco exposure found that 37 DNA CG sites in 30 genes of neonatal leukocytes have greater than 10% increase or 10% decrease in its CG methylation contents associated with parental tobacco smoke, and the CG methylation contents in certain genes' promoter have a significant interaction for the development of asthma and allergic rhinitis in childhood [6]. On the other hand, paternal influence on the development of asthma occurs in later childhood, suggesting genetic effects on the development of allergic sensitization are also important in children exposing to aeroallergens, pollution, and complementary foods beyond infancy. Fortunately, not all perinatal factors are risk to the development of childhood asthma, certain perinatal factors may contribute to protection from the development of asthma. Prenatal exposure to pets, vitamin D supplement, or antioxidants such as Mediterranean diet is shown to be protective from the development of asthma. In postnatal stage, breastfeeding, intake of probiotics, and growing up in farms [7] are associated with less risk to allergy. 

## 3. Future Perspectives

Another review article on whether perinatal diets such as vitamins, polyunsaturated fatty acid, protein-hydrolyzed infant formula, or the time and types of complementary foods are protective from the development of asthma is not included in this issue because of its complex controversy and the paper submitted for this issue was not good enough for publication. Neither the hygiene hypothesis for the development of asthma nor the persistence and remission of childhood asthma in adolescents are included in this issue. This guest editor team including K. D. Yang, MD, PhD, from the Department of Medical Research and Development, Show Chwan Health Care System, Taiwan; S.-K. Huang, PhD, from Johns Hopkins Asthma Center, Johns Hopkins University, Baltimore, USA; H.-J. Su, PhD, from Department of Environmental and Occupational Health, College of Medicine, National Cheng Kung University, Tainan, Taiwan; J. Abe, M.D., Ph.D., from the Department of Allergy and Immunology, National Research Institute for Child Health and Development, Tokyo, Japan anticipates that another issue of perinatal programming of asthma including advances in the knowledge of DOHaD depicting influence of nutrition and hygiene on the development and remission of asthma will come not far in order to provide a prospect for early prediction and prevention of childhood asthma.

## References

[1] Barker DJP, Osmond C (1986). Infant mortality, childhood nutrition, and ischaemic heart disease in England and Wales. *The Lancet*.

[2] Hales CN, Barker DJP (1992). Type 2 (non-insulin-dependent) diabetes mellitus: the thrifty phenotype hypothesis. *Diabetologia*.

[3] Ravelli ACJ, Van Der Meulen JHP, Osmond C, Barker DJP, Bleker OP (1999). Obesity in adults after prenatal exposure to famine. *American Journal of Clinical Nutrition*.

[4] Yang KD, Ou CY, Hsu TY (2007). Interaction of maternal atopy, CTLA-4 gene polymorphism and gender on antenatal immunoglobulin E production. *Clinical and Experimental Allergy*.

[5] Chen RF, Huang HC, Ou CY (2010). MicroRNA-21 expression in neonatal blood associated with antenatal immunoglobulin e production and development of allergic rhinitis. *Clinical and Experimental Allergy*.

[6] Yang KD Perinatal genetic and epigenetic programming of childhood asthma.

[7] Von Mutius E, Vercelli D (2010). Farm living: effects on childhood asthma and allergy. *Nature Reviews Immunology*.

